# Elevated Ictal Brain Network Ictogenicity Enables Prediction of Optimal Seizure Control

**DOI:** 10.3389/fneur.2018.00098

**Published:** 2018-03-01

**Authors:** Marinho A. Lopes, Mark P. Richardson, Eugenio Abela, Christian Rummel, Kaspar Schindler, Marc Goodfellow, John R. Terry

**Affiliations:** ^1^Living Systems Institute, University of Exeter, Exeter, United Kingdom; ^2^Wellcome Trust Centre for Biomedical Modelling and Analysis, University of Exeter, Exeter, United Kingdom; ^3^EPSRC Centre for Predictive Modelling in Healthcare, University of Exeter, Exeter, United Kingdom; ^4^Institute of Psychiatry, Psychology and Neuroscience, King’s College London, London, United Kingdom; ^5^Support Center for Advanced Neuroimaging (SCAN), University of Bern, Bern, Switzerland; ^6^Department of Neurology, Inselspital, Bern, Switzerland

**Keywords:** epilepsy surgery, ictogenic network, intracranial EEG, network dynamics, neural mass model

## Abstract

Recent studies have shown that mathematical models can be used to analyze brain networks by quantifying how likely they are to generate seizures. In particular, we have introduced the quantity termed brain network ictogenicity (BNI), which was demonstrated to have the capability of differentiating between functional connectivity (FC) of healthy individuals and those with epilepsy. Furthermore, BNI has also been used to quantify and predict the outcome of epilepsy surgery based on FC extracted from pre-operative ictal intracranial electroencephalography (iEEG). This modeling framework is based on the assumption that the inferred FC provides an appropriate representation of an ictogenic network, i.e., a brain network responsible for the generation of seizures. However, FC networks have been shown to change their topology depending on the state of the brain. For example, topologies during seizure are different to those pre- and post-seizure. We therefore sought to understand how these changes affect BNI. We studied peri-ictal iEEG recordings from a cohort of 16 epilepsy patients who underwent surgery and found that, on average, ictal FC yield higher BNI relative to pre- and post-ictal FC. However, elevated ictal BNI was not observed in every individual, rather it was typically observed in those who had good post-operative seizure control. We therefore hypothesize that elevated ictal BNI is indicative of an ictogenic network being appropriately represented in the FC. We evidence this by demonstrating superior model predictions for post-operative seizure control in patients with elevated ictal BNI.

## Introduction

Resective surgery is a treatment option for pharmacoresistant epilepsy patients. The paradigm of epilepsy surgery is to identify and remove the brain tissue responsible for the generation of seizures; the *epileptogenic zone* (1). The location of this tissue is inferred based on a qualitative analysis of different brain imaging modalities; specifically, MRI is used to detect epileptogenic lesions, and intracranial electroencephalography (iEEG) to find the seizure onset zone (2, 3). The prevailing hypothesis underpinning epilepsy surgery is that there is a *seizure focus* (1). However, it is becoming recognized that even presumed “focal” epilepsies can emerge from distributed *ictogenic networks* (4–6). This new understanding may explain in part why surgery is often unsuccessful and long-term positive outcome may be lower than 25% in extra-temporal cases (7, 8).

Building on these findings, mathematical methods have been proposed that interrogate clinical data to elucidate the ictogenic network and determine targets for surgery (9–13). In particular, recent methods have been developed based on functional connectivity (FC) networks derived from iEEG time series. In this case, nodes of the FC network represent brain regions in the vicinity of electrodes. Connections between nodes in the network are formed based on the presence of significant statistical associations between the signals recorded from two regions [see Ref. (14, 15) and references therein]. To understand the generation of seizures in these networks, and thereby determine targets for surgery, recent studies have placed mathematical models of seizure transitions onto the nodes of FC networks (9, 11, 13). Such models allow to simulate transitions between “normal” and “seizure-like” states, thereby capturing the “ictogenicity” of the network. We recently introduced means to quantify this effect, by measuring *brain network ictogenicity* (BNI), which is the propensity for nodes to generate seizure-like, rather than “normal,” activity. In practice, BNI can be calculated as the average proportion of time that network nodes spent in the seizure-like state during a sufficient long simulation time. BNI has been shown to be a useful biomarker of idiopathic generalized epilepsy (16–19) and a predictor of post-surgical seizure control (9, 13).

In pre-surgical planning, intracranial electrodes are sometimes implanted to test hypotheses regarding the location of the epileptogenic zone (2). The implantation strategy is informed by clinical EEG and neuroimaging data (2), but it is plausible that in some cases iEEG electrodes do not adequately sample from the ictogenic network. It is important to note that iEEG has a number of limitations, including limited precision for deep targets, confined tissue coverage, and it is constrained to avoid morbidities (20). In addition, since the iEEG signals vary over time, inferred functional connections vary depending on the state of the brain. For example, analysis of peri-ictal time courses of iEEG recordings have demonstrated variation in FC topologies during seizures compared with pre- and post-ictal time periods (21–24). However, at present, we have no *a priori* techniques to determine whether FC extracted from iEEG recordings, which depends on the placement of electrodes and the underlying brain state, is an appropriate representation of the underlying ictogenic network.

To address this, we study how BNI derived from iEEG signals evolves over peri-ictal epochs. We measure BNI across peri-ictal iEEG recordings from 16 patients suffering from pharmacoresistant epilepsies who underwent epilepsy surgery. We find that on average BNI increases from the pre-ictal to the ictal state and declines thereafter. However, we find considerable variability in this trend at the individual level, with not all individuals showing elevated ictal BNI. We further show that elevated ictal BNI is preferentially observed in patients who had good sustained post-operative seizure control, i.e., who were free or almost free of disabling seizures after surgery. We therefore propose that FC displaying elevated ictal BNI is an appropriate representation of the ictogenic network. Consequently, we test whether our previously reported modeling framework to predict epilepsy surgical outcome (9) can be optimized if we restrict analysis to cases which reveal increased ictal BNI.

## Materials and Methods

### Clinical iEEG Recordings and FC

We studied iEEG from 16 subjects who underwent epilepsy surgery at Inselspital Bern (11 females, mean age 31, and median post-surgical follow-up 3 years). The surgery outcome was measured in terms of seizure rate using the Engel scale (see Table S1 in Supplementary Material). The signals were recorded intracranially by grid, strip, and depth AdTech electrodes (WI, USA) and a NicoletOneTM recording system (VIASYS Healthcare Inc., WI, USA). This study was approved by the Internal Review Board of the Inselspital (approval No. 159399, dated November 26, 2013). All patients gave written informed consent that imaging and EEG data may be used for research purposes. Other details about this dataset have been previously described (9, 24).

**Figure 1:**
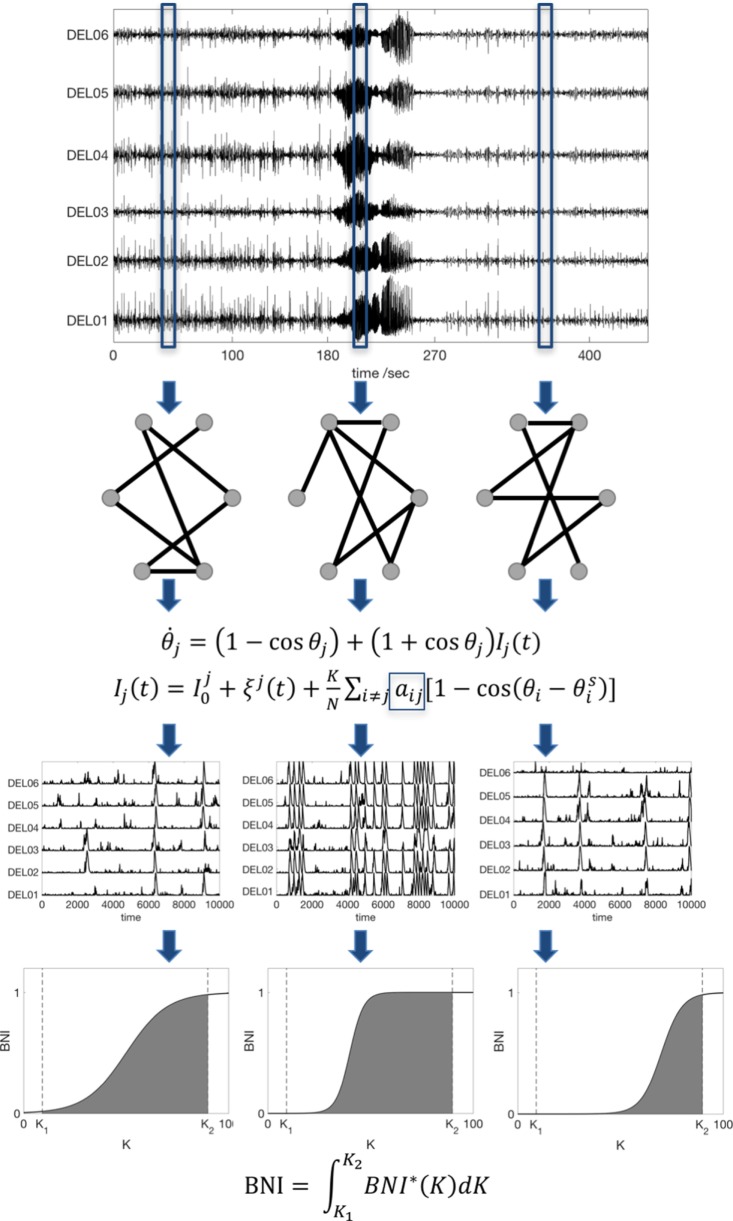
Scheme of the data analysis procedure. An iEEG peri-ictal recording is divided into *N* segments, and each one is used to infer a functional connectivity network based on a surrogate-corrected cross-correlation measure. We then compute the dynamics in each network using the theta model, and we find the *BNI* as function of the coupling *K*. Finally, to avoid an arbitrary choice of *K*, the BNI is redefined as the integral of *BNI* in the interval [*K*_1_, *K*_2_]. For comparison between networks, the interval [*K*_1_, *K*_2_] is fixed, and sufficiently large to account for the variation of *BNI* as function of *K*. Note that the actual recordings comprise tens of channels; hence, the actual networks are much larger than the ones represented here.

For our analysis, we considered two peri-ictal epochs per patient each comprising 3 min of pre-ictal activity, a seizure, and 3 min of post-ictal activity, as visually determined by an experienced epileptologist (Kaspar Schindler). The iEEG signals were down-sampled to 512 Hz and re-referenced against the median of all artifact-free segments as judged by visual inspection (Kaspar Schindler). These data were then band-pass filtered between 0.5 and 120 Hz and notch-filtered between 48 and 52 Hz. Each epoch was divided into a set of 8 s segments, with 1 s gaps between each of them. The gaps were pragmatically chosen to be 1 s to provide a balance between the need for sufficient sampling and computational tractability. Next, we computed 10 univariate iterated amplitude adjusted Fourier transform surrogates for each segment. The segments were then divided in 10 subsegments of 2 s distributed with minimal overlap. This procedure resulted in an ensemble of 10 subsegments for the original time series, and 100 subsegments for the surrogates. The FC was inferred from the correlations between the time series of each iEEG channel using the Pearson’s equal-time (zero-lag) cross-correlation coefficient ρ. We used the Mann–Whitney–Wilcoxon *U*-test to assess whether the correlations in the original time series (ρ_0_) were significantly different compared with the correlations in the surrogates (ρ_surr_), and we applied Bonferroni–Holm corrections to account for multiple comparisons. We thus obtained a surrogate-corrected correlation matrix using the formula:

$$C=\frac{\rho_{0}-\rho_{\text{surr}}}{1-\rho_{\text{surr}}}s$$

where *s* = 1 if the null hypothesis of the statistical test was rejected, or *s* = 0 otherwise (13, 24). The first and second rows in Figure 1 illustrate our methods. To test the robustness of the results, we also inferred FC using surrogate-corrected non-linear *h*^2^ index (25), i.e., using the same methods as above using the *h*^2^ index instead of the Pearson cross-correlation for the calculation. In this case, we obtained the surrogate-corrected *h*^2^ index matrix,

$$H=h^{2}s.$$

### Mathematical Model

To study the time evolving FC across peri-ictal epochs, we modeled network dynamics on each FC network. We considered the nodes as interacting neural masses, which we approximate using the theta model, as previously described (13). In this model, the dynamic state of each node is described by its phase θ*_j_* and it obeys the ODE:

$${\dot{\theta }}_{j}=(1-\text{cos}\theta_{j})+(1+\text{cos}\theta_{j})I_{j}(t),$$

where *I_j_*(*t*) accounts for incoming currents,

$$I_{j}(t)=I_{0}^{j}+\xi^{j}(t)+\frac{K}{N}\underset{i\neq j}{\sum }a_{ij}[1-\text{cos}(\theta_{i}-\theta_{i}^{s})],$$

the index *j* denotes node *j* (*j* = 1, 2, …, *N*, where *N* is number of nodes, i.e., of electrodes), $I_{0}^{j}+\xi^{j}(t)$ represents noisy inputs (Gaussian noise), *K* is the global scaling coupling, *a_ij_* is the *i*,*j*th entry of the adjacency matrix (the FC network), and $\theta_{i}^{s}$ is the steady state of node *i* (13). Each node is able to transit between a “normal state” (a stable fixed point), and a “seizure state” (a stable limit cycle) through a saddle-node on invariant circle (SNIC) bifurcation. The third row of Figure 1 shows representative model dynamics, in which the spiking activity corresponds to the seizure state.

We quantified network dynamics using *BNI*: the average of the fraction of time that each node spends in the seizure state (9, 13, 18). This fraction is computed by integrating the differential equation for a sufficiently long time (we used 4 × 10^6^ time steps) and evaluating the time spent in spiking dynamics. Spikes were extracted by applying a threshold to the phases as described in Ref. (13). Since, for a given network, *BNI* varies upon the particular choice of the global coupling parameter, *K* (see the fourth row of Figure 1), to avoid an arbitrary choice of *K*, we redefine BNI as

$$\text{BNI}=\overset{K_{2}}{\underset{K_{1}}{\int }}BNI^{*}(K)dK\text{.}$$

A comparison of BNI between two networks is only meaningful if all model parameters are the same and *K*_1_ and *K*_2_ are chosen such that BNI* varies in the interval 0–1. To enable comparison between individuals, BNI was normalized by the maximum BNI within each peri-ictal epoch. Also, to render the temporal evolution of BNI over the peri-ictal period similar in all patients, the ictal duration was normalized so that 10 BNI values were calculated for each patient in this epoch. We emphasize that BNI characterizes the underlying FC network inferred from the data by measuring the propensity of the network to generate seizures *in silico*. The measure is only meaningful, however, when used to compare different networks for fixed model parameters. Figure 1 summarizes our methods.

Surgical resection was modeled as the removal of *n_s_* nodes from the network. The impact on *BNI* after node removal was measured by

$$\Delta BNI^{ns}=\frac{BNI_{\text{pre}}-BNI_{\text{post}}^{ns}}{BNI_{\text{pre}}}$$

where *BNI*_pre_ is a reference state of the pre-surgery network (parameters were chosen such that *BNI*_pre_ = 0.5) and $BNI_{\text{post}}^{ns}$ is *BNI* upon removal of the *n_s_* nodes. In the model, a successful surgery will have higher Δ*BNI*^ns^ than an unsuccessful one. The set *n_s_* of nodes removed were identified from the coregistration of high resolution T1-weighted MR images acquired before and after surgery in all individuals [see the full details of these procedures in Ref. (24)]. We made use of the Δ*BNI^ns^* as computed by Goodfellow et al. (12) to quantify the outcome of the actual surgeries that were performed on the 16 subjects. We note that Goodfellow et al. utilized the Wendling model, which has recently been shown to be equivalent to the theta model for the purpose of modeling surgical resections (13). Furthermore, in this case, the Δ*BNI^ns^* was measured using FC inferred based on mutual information from the first half of the seizures [as these correlate well with the epileptogenic tissue (24)].

### Statistical Analysis

To test for statistically significant differences between ictal BNI and pre- and post-ictal BNI of the whole cohort of patients, we used the Kruskal–Wallis test. Differences were considered statistically significant when the *p*-values were less than 0.05. We then analyzed patients individually and used the Mann–Whitney–Wilcoxon *U*-test to assess whether the median ictal BNI was higher than the pre-ictal BNI. A chi-square test was performed to test whether the apparent correlation between elevated ictal BNI and surgical outcome was statistically significant. We generated 10,000 random BNI classifications and estimated how likely was to find the observed relation between BNI and surgical outcome by chance. In addition, we evaluated the ability of Δ*BNI^ns^* to predict the post-operative outcome through the area under the curve (AUC) of the receiver operating characteristic. Having obtained two AUC, we wanted to compare them. We used all possible combinations of half-group splitting of Δ*BNI^ns^* to find a distribution of AUC values (i.e., two distributions, one for each AUC). We then compared the two distributions using the Mann–Whitney–Wilcoxon *U*-test.

## Results

**Figure 2:**
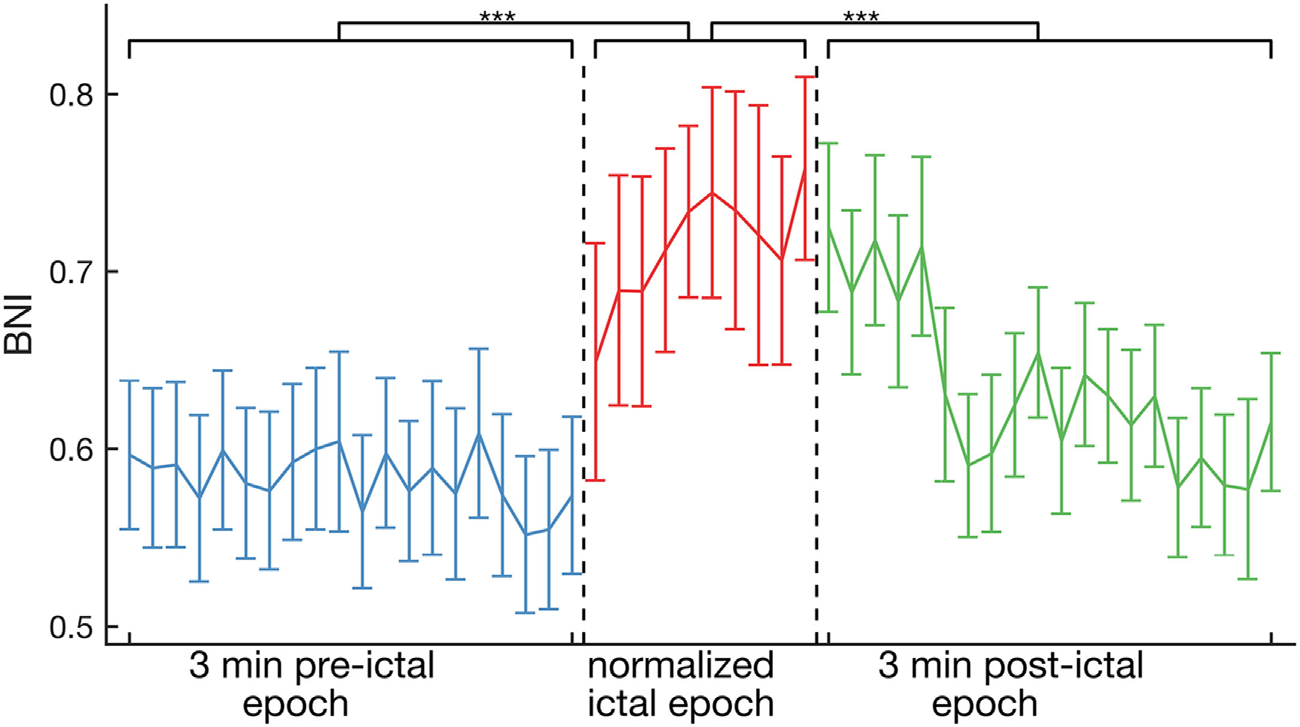
Average across the whole cohort of 16 patients of BNI as a function of time. On average, BNI during the ictal epoch is higher than for pre- and post-ictal epochs (*p* < 0.001, Kruskal–Wallis test). The ictal epochs were all normalized to 10 BNI values for comparison. The error bars account for the variability between peri-ictal epochs and patients.

Figure 2 shows the evolution of average BNI from peri-ictal recordings of all 16 individuals. BNI was found to be higher on average in the ictal epoch, relative to the pre- and post-ictal epochs (*p* < 0.001, Kruskal–Wallis test). Equivalent results were also observed for FC inferred using the non-linear *h*^2^ index (see Figure S1 in Supplementary Material). Specifically, we observed a sharp increase in BNI at seizure onset, but, interestingly, BNI remained at similar levels to those of the ictal epoch for about 1 min into the clinically defined post-ictal epoch. These results were robust for different choices of the strength of coupling between nodes in the BNI calculation (see Figure S2 in Supplementary Material).

**Figure 3:**
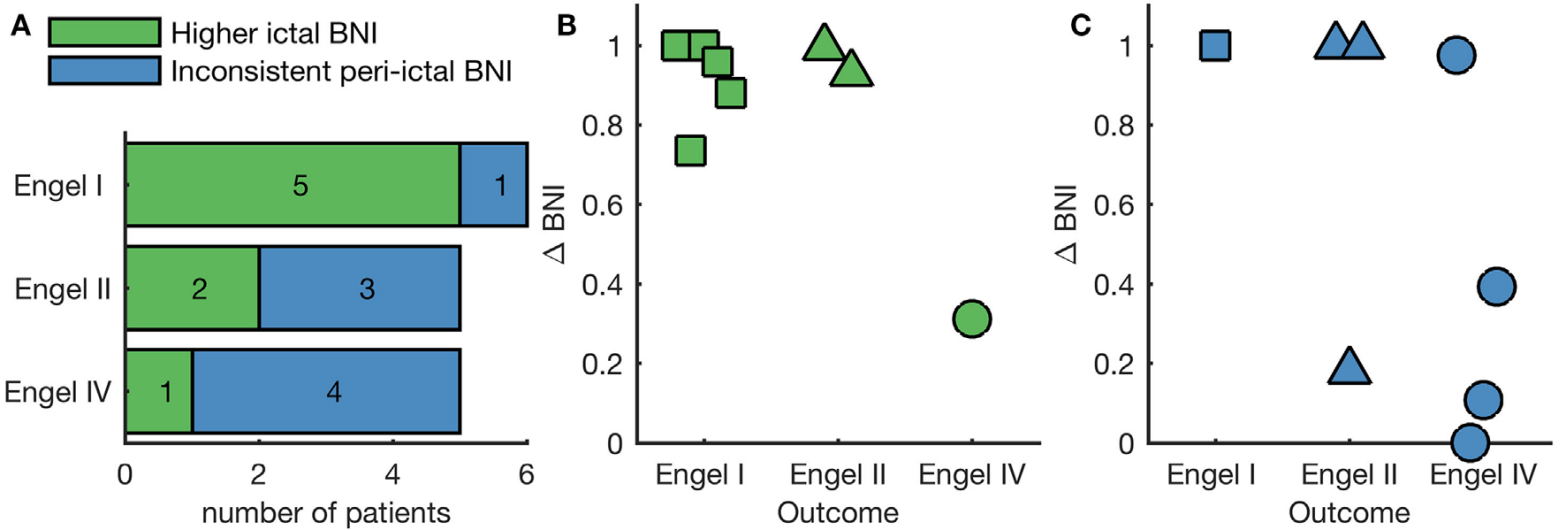
Individual analysis of BNI as function of surgical outcome. **(A)** Number of patients within each Engel class that either present a statistically significant higher ictal BNI compared with pre-ictal BNI in both peri-ictal epochs independently (green bars) or do not (blue bars). Panels **(B,C)** show the quantification of surgical outcome with higher ictal and inconsistent peri-ictal BNI, respectively. Each marker is the Δ*BNI*^ns^ of a different patient. Here, high values of Δ*BNI*^ns^ are effectively model predictions of a good surgical outcome, whereas low values predict negative surgical outcome. In panel **(B)**, Δ*BNI*^ns^ correctly classifies all patients displaying higher ictal BNI, whereas in panel **(C)** two patients are incorrectly classified.

We next examined the pattern of BNI evolution for each of the two peri-ictal epochs of each individual separately. A one-tailed Mann–Whitney–Wilcoxon *U*-test was performed to assess whether the median BNI in the ictal period was larger than the pre-ictal median BNI. We found elevated ictal BNI in both peri-ictal epochs of eight individuals, with the remaining eight individuals not displaying a significant increase in BNI during the ictal period in at least one of the peri-ictal epochs. Figure S3 in Supplementary Material shows the BNI evolution of four representative individuals, two exhibiting consistent elevated ictal BNI in both peri-ictal epochs, and two presenting inconsistent peri-ictal BNI. We further found that elevated ictal BNI was correlated with post-operative outcome (*p* = 0.049, chi-square test comparing to 10,000 random BNI classifications). In five of six people who were seizure free post-surgery (Engel I), we observed a significant increase in BNI in ictal epochs relative to the pre-ictal epochs (Figure 3A). On the other hand, four of five patients who had no improvement post-surgery (Engel IV) showed no such significant increase in BNI in either one or both seizures (inconsistent peri-ictal BNI in Figure 3A). We hypothesized that elevated ictal BNI implies that the inferred FC appropriately characterizes the ictogenic network; therefore, methods to predict surgical outcome based on FC would be optimal when restricted to these cases. To test this hypothesis, we focused on the eight individuals displaying elevated ictal BNI. Figure 3B shows the Δ*BNI^ns^* of these individuals, where *n*_s_ corresponds to nodes resected in the actual surgery. Δ*BNI^ns^* represents a model prediction for the change in seizure frequency due to the performed resection; high values of Δ*BNI^ns^* indicate that removal of resected nodes dramatically reduces the rate of seizures in the model, whereas low values of Δ*BNI^ns^* indicate a smaller reduction in the model seizure rate [see Ref. (9, 24) for more details about the identification of the *n_s_* nodes]. In the eight cases with elevated ictal BNI, we found that the model was capable of correctly predicting the surgical outcome; we observed higher values of Δ*BNI^ns^* for good, compared with poor outcome cases. Figure 3C displays the Δ*BNI^ns^* of the subjects with inconsistent peri-ictal BNI, showing misclassification of two individuals: an Engel class II had low Δ*BNI^ns^*, whereas an Engel class IV had high Δ*BNI^ns^*. AUC is significantly lower in Figure 3C compared with Figure 3B (AUC = 0.87 and AUC = 1, respectively; *p* < 0.001, Mann–Whitney–Wilcoxon *U*-test calculated on the distribution of all AUC using half-group splitting).

## Discussion

To date, the concept of BNI—that is the time spent in the seizure state averaged across each node within a network—has proved to be valuable for both epilepsy diagnosis (16–19) and assessment of epilepsy surgery (9, 13). Building on this work, similar findings have been observed using an alternative computer modeling framework (11), in which the authors studied pre-surgical functional networks to infer the pathological nodes, rather than estimating the result of the actual surgical procedures on the network (9). Herein, we propose a method that utilizes BNI to evaluate how well FC characterizes the ictogenic network. We studied a dataset comprising iEEG from 16 individuals who underwent pre-surgical monitoring, finding that BNI is elevated in ictal epochs at the group level (Figure 2). Furthermore, by assessing individual subjects, we found that 8 of 16 individuals display an elevated ictal BNI (Figure 3A). Presuming these cases are those for which FC has appropriately characterized the ictogenic network, we should expect optimal model-based predictions in these cases. Figure 3B illustrates that our modeling framework (9) is capable of correctly classifying the outcome of all individuals displaying elevated ictal BNI. Effectively, and taking into account Figure 3C, this suggests that observing elevated ictal BNI is a sufficient, but not necessary, condition under which model predictions are optimal and therefore might guide surgical decision making.

It has previously been shown that FC topology changes during seizures (21–24). Here, we present the first demonstration of the effect that these changes have on BNI. Figure 2 further shows that BNI remains high during the first minute of the post-ictal epoch. This effect may derive from previously observed dynamic reorganization of spatial correlation, with elevated correlation after seizure termination (26). For future work, we plan to test whether individuals who suffer from clusters of seizures might have a higher post-ictal BNI than individuals who do not. We note, however, that high post-ictal BNI is not observed when FC is inferred using the *h*^2^ index (Figure S1 in Supplementary Material). Future work will also therefore investigate the ways in which linear and non-linear channel associations evolve at seizure termination.

The emerging field of network model-based recommendations for epilepsy surgery is based on a number of assumptions. First, it assumes the existence of an ictogenic network, i.e., a brain network responsible for the generation of seizures. In fact, there is extensive neuroimaging and iEEG evidence for the widespread involvement of brain regions in focal epilepsies (4–6, 27). However, within such a network, there is the potential for individual nodes to drive seizure activity, since seizures can emerge due to both intrinsic tissue properties and connections within a larger network (28, 29). The second assumption is that the implanted electrodes were placed in such a way that inferred FC appropriately reflects (or covers) the ictogenic network. Electrode placement is a clinical decision based on interpretation of other neuroimaging data (e.g., MRI and scalp EEG) and seizure semiology, not typically considering large-scale network properties. As a consequence, this assumption could, in some instances, be invalid. It has been suggested that electrodes should preferentially be placed close to the seizure onset zone (24), but it remains unclear how to optimize the characterization of the ictogenic network. The approach we propose could be further developed to study non-invasive neuroimaging modalities to guide optimal electrode placement to facilitate a patient-specific analysis. The third assumption concerns the model itself. Here, we assume that a stable fixed point, a limit cycle, and a SNIC bifurcation are a sufficient description of the “normal state,” “seizure state,” and the “transition mechanism” between these two states. Other studies have considered different mechanisms. For example, Sinha et al. (11) considered a subcritical Hopf bifurcation as the transition mechanism. Future studies should compare model predictions when these conditions are varied. Finally, we do not consider post-surgical brain network plasticity. Such long-term structural evolution may be paramount to understanding long-term surgical outcomes, particularly reasons underlying early and late seizure recurrences (8). While the validity of these assumptions remains an open question, we showed here that model-based predictions of surgical outcome are optimal if individuals display elevated ictal BNI consistently across different peri-ictal epochs (see Figure 3). We thus interpret that elevated ictal BNI is an indication of successfully resolving the ictogenic network for the given choice of electrode placement, FC inference method, and mathematical model.

## Ethics Statement

This study was carried out in accordance with the recommendations of the Internal Review Board of the Inselspital with written informed consent from all subjects. All subjects gave written informed consent in accordance with the Declaration of Helsinki. The protocol was approved by the Internal Review Board of the Inselspital (approval No. 159399, dated November 26, 2013).

## Author Contributions

ML, MG, and JT: study concept and design, results interpretation, and manuscript drafting and revision. ML: data analysis. MR: results interpretation and manuscript revision. EA, CR, and KS: acquisition of clinical data, results interpretation, and manuscript revision.

## Conflict of Interest Statement

The authors declare that the research was conducted in the absence of any commercial or financial relationships that could be construed as a potential conflict of interest.
