# Supplementary material for: Elevated Ictal Brain Network Ictogenicity Enables Prediction of Optimal Seizure Control
Source: Front Neurol. 2018 Mar 1;9:98. doi: 10.3389/fneur.2018.00098 (PMC5837986; doi:10.3389/fneur.2018.00098)
Supplement: Supplementary file 1 [file data_sheet_1.docx]

Supplementary Material

Elevated Ictal Brain Network Ictogenicity Enables Prediction of Optimal Seizure Control

Marinho A. Lopes^*^, Mark P. Richardson, Eugenio Abela, Christian Rummel, Kaspar Schindler, Marc Goodfellow, John R. Terry

*** Correspondence:** Marinho A. Lopes: m.lopes@exeter.ac.uk

# Supplementary Figures and Tables

**1.1 Supplementary Figures**


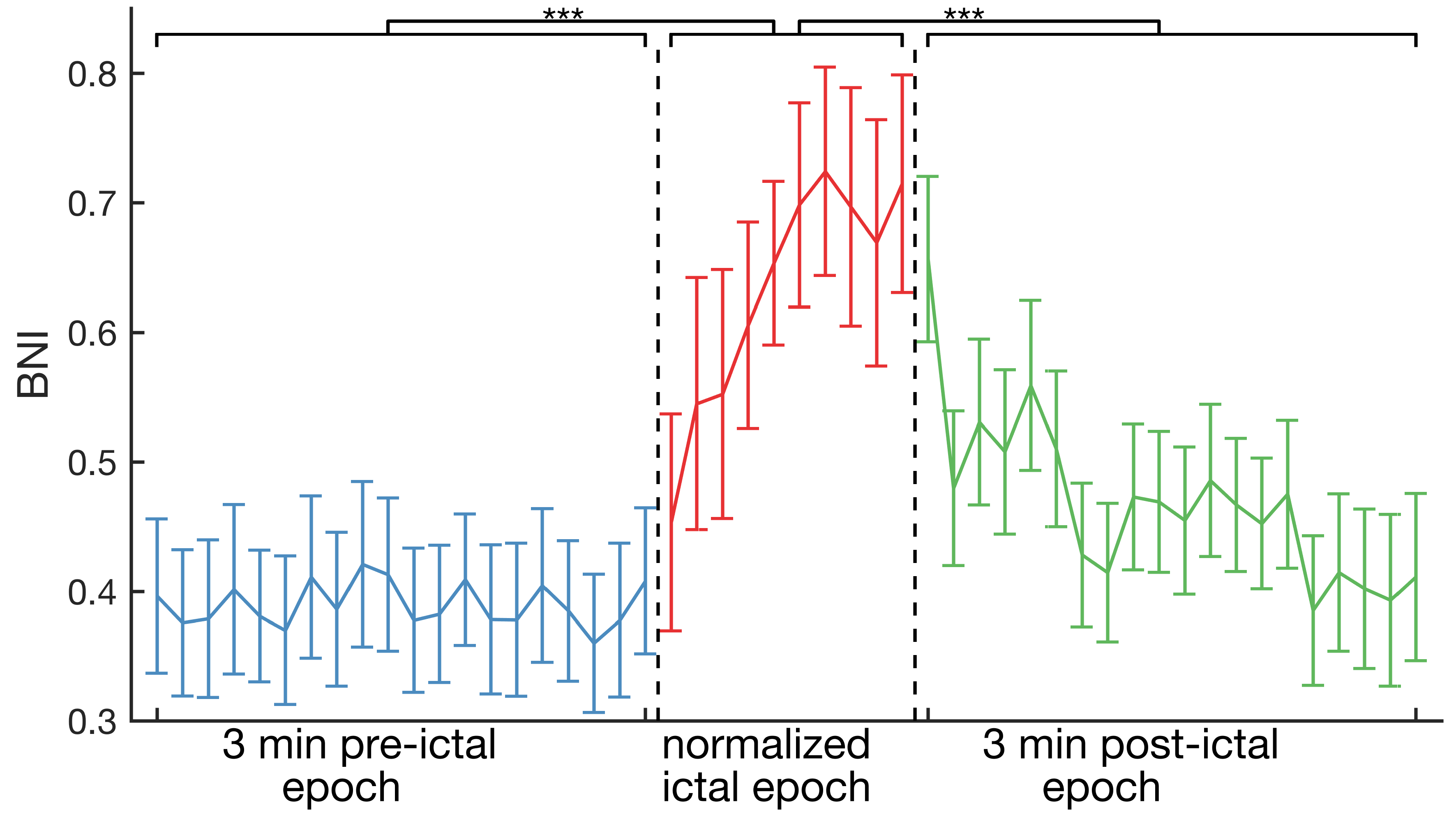


**Supplementary Figure 1.** BNI as function of time for the whole cohort of 16 patients (2 peri-ictal epochs per patient). The BNI was computed on surrogate-corrected nonlinear $h^{2}$index FC. The ictal epoch has a higher BNI compared to the pre- and post-ictal epochs ($p<0.0001$, Mann-Whitney-Wilcoxon U-test). The different colors distinguish the different epochs. The error bars account for the variability between peri-ictal epochs and patients. The duration of the ictal-epochs was normalized to $10$ points for comparison as in Figure 2.

**
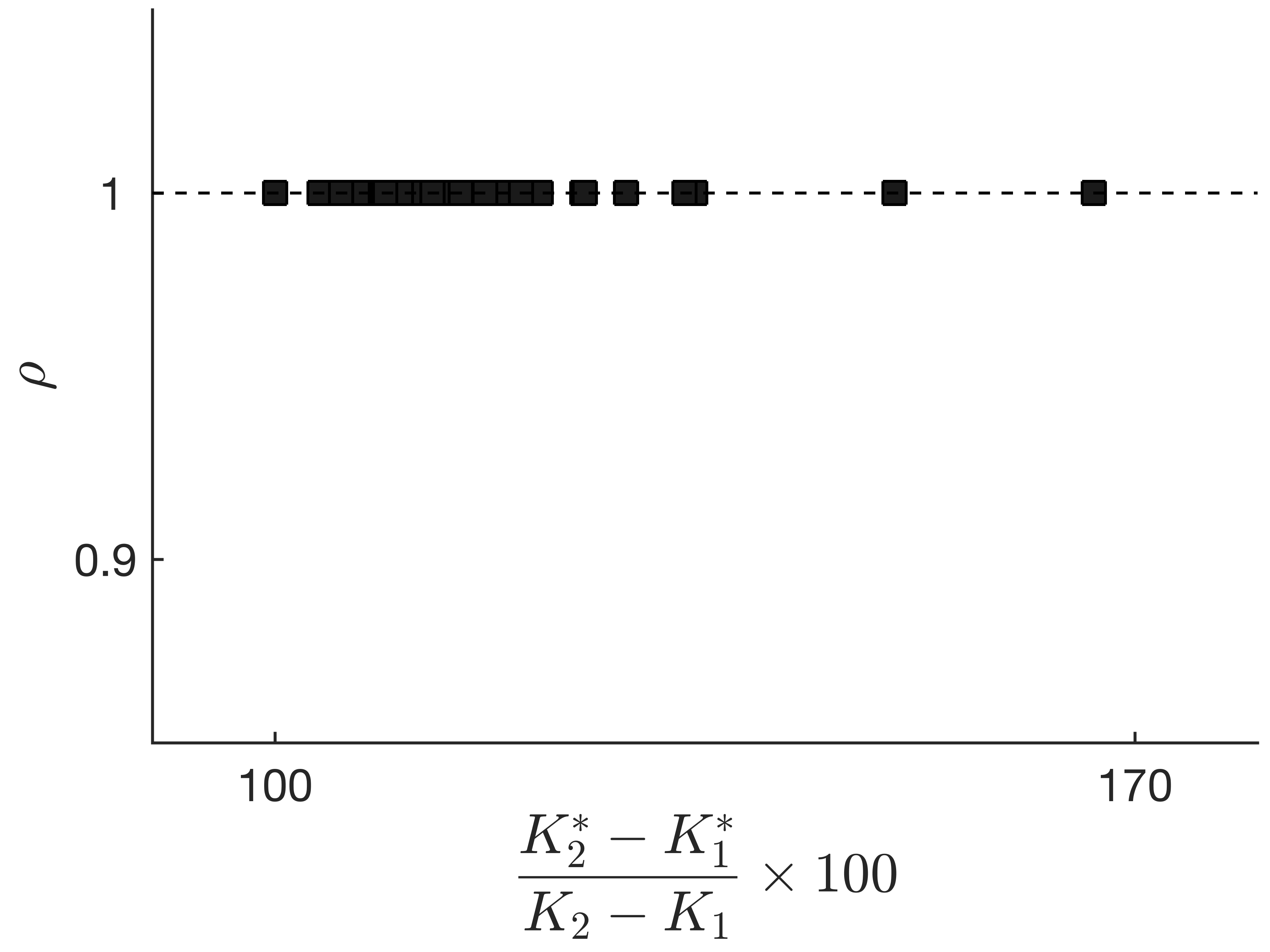
**

**Supplementary Figure 2.** Robustness of the BNI to the choice of the range of coupling. The results presented in Figure 2 were compared to equivalent computations of BNI using larger ranges of coupling ($K_{2}^{*}-K_{1}^{*}$) and the robustness was assessed using Pearson correlation ($\rho$). The comparison was performed for each and every patient and seizure. The results are robust ($min\{\rho\}>0.99$).


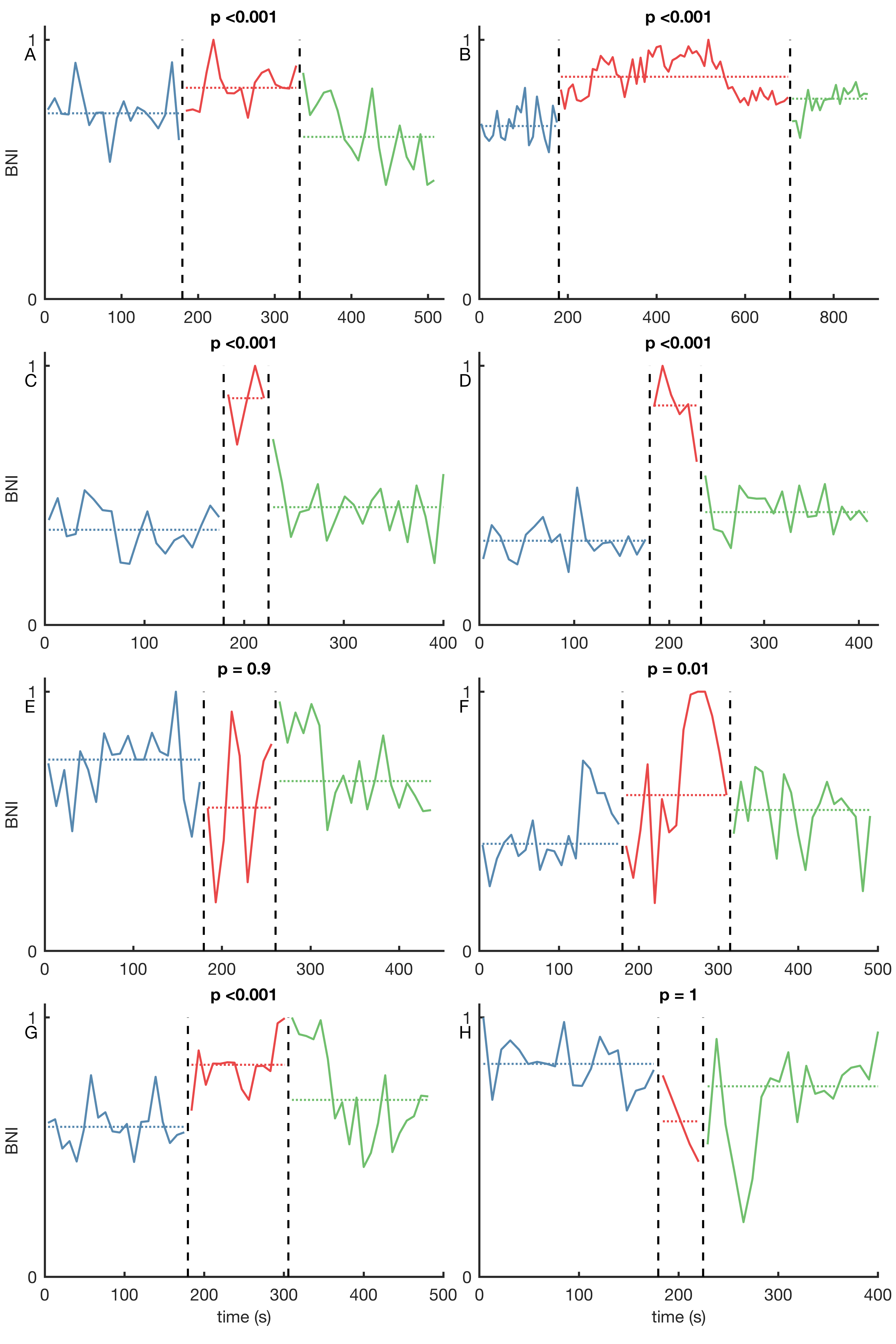


**Supplementary Figure 3.** BNI as function of time for four representative individuals. Each row corresponds to a different individual and each column to a different peri-ictal epoch, i.e., the figure shows two peri-ictal epochs per individual. As in Figure 2, the pre-ictal epochs are the blue lines, the ictal epochs are the red lines, and the post-ictal epochs are the green lines. The dotted lines represent the median BNI in each epoch. BNI was normalized such that the maximum BNI in each epoch is 1. The p-values indicate whether the median ictal BNI is higher than the median pre-ictal BNI (Mann-Whitney-Wilcoxon U-test). We consider that an individual has a consistent higher ictal BNI if the median ictal BNI is higher than the median pre-ictal BNI in both peri-ictal epochs. According to this criterion, the individuals represented in panels A-D show consistent higher ictal BNI, whereas the other individuals represented in panels E-H present inconsistent peri-ictal BNI. The individuals represented in panels A, B and E, F had a positive surgical outcome (Engel I), and the individuals represented in panels C, D and G, H had a negative surgical outcome (Engel IV).

**1.2 Supplementary Tables**

**Supplementary Table 1.** Detailed patient information. Patients 2, 3, 4, 5, 6, 9, 10, and 12 showed higher ictal BNI.

| Patient | Engel class | Gender | Age (y) | Syndrome | Hemisphere | Lesion MRI visible | Total No.  of iEEG  channels | No. of artifact  free iEEG  channels | No. of resected iEEG  channels | Resection type | Follow up (y) |
| --- | --- | --- | --- | --- | --- | --- | --- | --- | --- | --- | --- |
| 1 | I | F | 26 | MTLE | R | y (hippocampal sclerosis) | 64 | 64 | 20 | amygdalo-hippocampectomy | 3 |
| 2 | I | F | 48 | MTLE | L | y (hippocampal sclerosis) | 64 | 64 | 13 | amygdalo-hippocampectomy | 3 |
| 3 | I | M | 27 | LTLE | L | n | 56 | 56 | 5 | temporo-lateral resection | 1 |
| 4 | I | M | 36 | PLE | L | y (pilocytic astrocytoma) | 77 | 74 | 6 | lesionectomy | 5 |
| 5 | I | F | 19 | MTLE | L | y (hippocampal sclerosis) | 44 | 42 | 11 | amygdalo-hippocampectomy | 5 |
| 6 | I | F | 25 | FLE/TLE | R | n | 104 | 98 | 11 | temporo-polar resection | 4 |
| 7 | II | F | 49 | FLE | R | y (focal cortical dysplasia) | 102 | 92 | 8 | lesionectomy | 4 |
| 8 | II | F | 46 | LTLE | R | n | 102 | 100 | 13 | amygdalo-hippocampektomie | 3 |
| 9 | II | M | 20 | LTLE | R | n | 79 | 54 | 14 | temporo-polar resection | 3 |
| 10 | II | M | 31 | LTLE | L | y (hippocampal sclerosis) | 74 | 59 | 17 | temporo-lateral resection | 3 |
| 11 | II | F | 24 | LTLE | L | n | 50 | 47 | 24 | temporo-polar resection | 3 |
| 12 | IV | F | 38 | LTLE | L | n | 62 | 59 | 2 | temporo-lateral resection | 4 |
| 13 | IV | F | 23 | LTLE | L | n | 63 | 61 | 10 | temporo-lateral resection | 2 |
| 14 | IV | F | 59 | MTLE | L | y (space occupying amygdala) | 52 | 49 | 8 | lesionectomy | 4 |
| 15 | IV | M | 32 | PLE | L | y (focal cortical dysplasia) | 98 | 62 | 4 | lesionectomy | 2 |
| 16 | IV | F | 31 | FLE | R | y (tuberous sclerosis) | 37 | 36 | 3 | lesionectomy | 2 |
